# Rapid Onset of Iron Overload Cardiomyopathy in Cirrhosis

**DOI:** 10.1016/j.jaccas.2026.108338

**Published:** 2026-05-13

**Authors:** Cyrus M. Nouraee, William H. Swain, David M. Harmon, Patrick S. Kamath, Margaret M. Redfield, Jody C. Olson

**Affiliations:** a Department of Internal Medicine, Mayo Clinic, Rochester, Minnesota, USA; b Department of Cardiovascular Medicine, Mayo Clinic, Rochester, Minnesota, USA; c Division of Gastroenterology and Hepatology, Mayo Clinic, Rochester, Minnesota, USA

**Keywords:** cardiac magnetic resonance imaging, cirrhosis, hepatic iron overload, iron overload cardiomyopathy (IOC), myocardial iron deposition, T2∗ mapping

## Abstract

Iron overload cardiomyopathy (IOC) is a rare cause of heart failure classically associated with hereditary or transfusion-related hemochromatosis. Although iron accumulation occurs in cirrhosis, the development and clinical course of IOC in this population remain poorly described. We report 2 patients with cirrhosis who developed IOC without hereditary hemochromatosis or transfusion-dependent anemia. Patient #1 was a 54-year-old man with alcohol-associated cirrhosis who developed severe cardiomyopathy (left ventricular ejection fraction 23%, 55% 1 year earlier). Patient #2 was a 56-year-old man with cryptogenic cirrhosis who presented with new cardiomyopathy (left ventricular ejection fraction 38%, 71% 9 months earlier). Both patients had elevated ferritin and transferrin saturation, along with reduced myocardial T2∗ relaxation time on cardiac magnetic resonance imaging, consistent with IOC. Both received iron chelation therapy; one patient died, and the other is awaiting combined heart-liver transplantation. These cases highlight IOC as an important consideration in patients with cirrhosis presenting with new cardiomyopathy.

## Visual Summary

**Figure:**
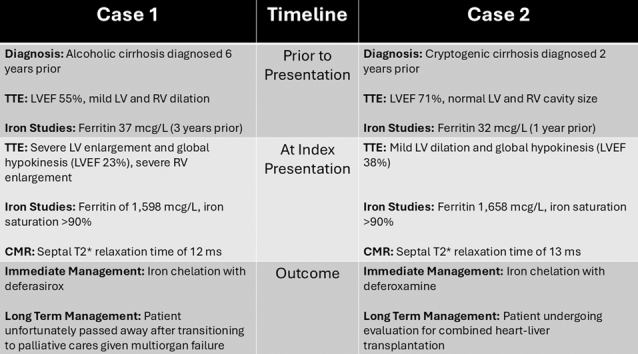

**Visual Summary:**
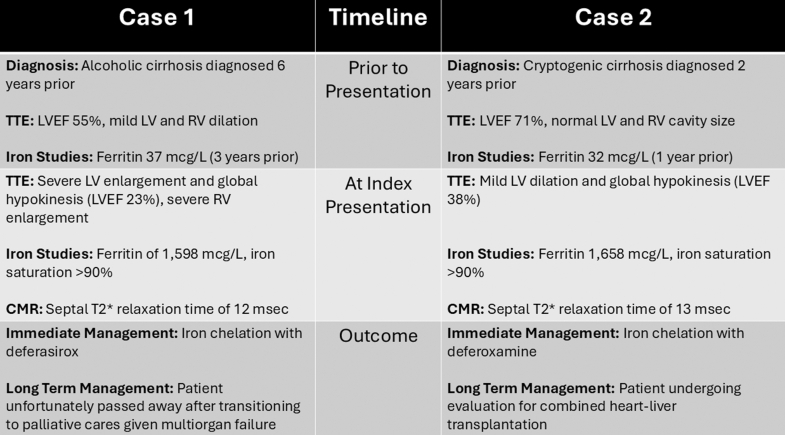
Clinical Course, Laboratory, and Imaging Findings of Patients With Iron Overload Cardiomyopathy Secondary to Cirrhosis CMR = cardiac magnetic resonance imaging; LV = left ventricular; LVEF = left ventricular ejection fraction; RV = right ventricular; TTE = transthoracic echocardiogram.

Iron overload cardiomyopathy (IOC) is a rare etiology of heart failure and is associated with a poor prognosis, resulting from excess iron deposition within the myocardial tissue, resulting in systolic and/or diastolic dysfunction independent of other etiologies of cardiomyopathy. It most commonly presents with diastolic dysfunction progressing to a dilated phenotype (∼90% of cases) with left ventricular (LV) remodeling and reduced LV ejection fraction (LVEF), while a restrictive phenotype is less common (<10%) and characterized by preserved ejection fraction, restrictive filling, pulmonary hypertension, and right heart dilation.^1^

**Take-Home Messages**

• Iron overload cardiomyopathy should be considered in patients with cirrhosis who present with new or rapidly progressive cardiomyopathy.
• Cardiac magnetic resonance imaging with T2∗ mapping may be useful for diagnosis and may have important implications for management and transplant evaluation in this patient population.

A transthoracic echocardiogram (TTE) may identify the consequences of iron overload on myocardial function, but it does not accurately quantitate myocardial iron concentration. Cardiac magnetic resonance imaging (CMR) remains the gold standard, with reduced myocardial T2∗ relaxation time (<20 ms) indicating cardiac siderosis in the appropriate clinical context.

IOC most often arises in systemic iron overload states such as hereditary hemochromatosis (HH) or transfusion-dependent anemias, which lead to progressive multiorgan hemosiderosis.^2^ However, hemosiderosis can also occur in the setting of cirrhosis because of reduced hepcidin levels secondary to advanced liver disease.^3^^,^^4^ This ultimately results in iron accumulation in multiple organs. Despite the awareness of this mechanism, clinical IOC in patients with cirrhosis is incompletely described in the current literature. Herein, we present 2 cases of rapid-onset IOC in patients with non-HH cirrhosis who were awaiting liver transplantation, highlighting a critical gap in the awareness and management of this population.

## Case 1

A 54-year-old man presents to the emergency department with a 27-lb weight gain and dyspnea. He has a history of alcohol-associated cirrhosis that was diagnosed 6 years before this presentation and is currently undergoing outpatient evaluation for liver transplantation. He had been abstinent from alcohol for the previous 5 years and had no known history of cardiac disease. One year before presentation, a routine pretransplantation TTE showed an LVEF of 55% with mild LV and right ventricular (RV) dilation and normal LV diastolic function. A coronary angiogram obtained 1 year prior revealed mild nonobstructive coronary artery disease.

**Figure 1:**
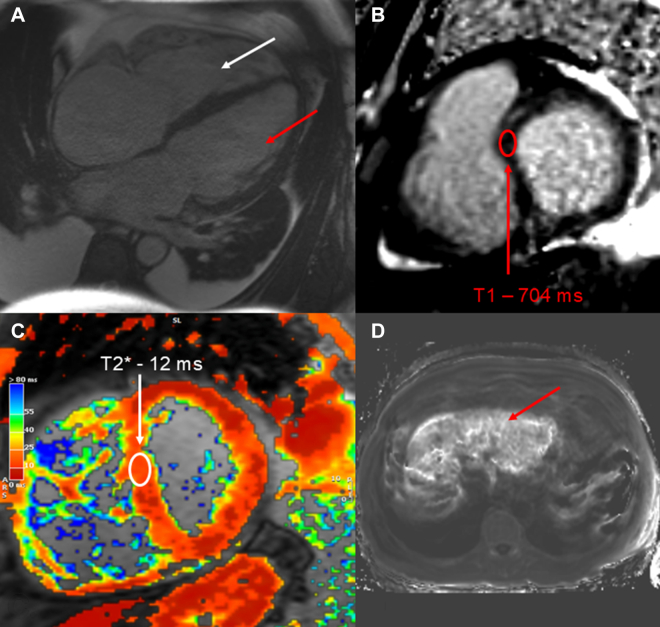
Cardiac and Liver Magnetic Resonance Imaging Findings of Patient Case 1 (A) Four-chamber view on cardiac magnetic resonance imaging (T1 weighted) showing severe biventricular (white arrow points to the right ventricle and red arrow to the left ventricle) and biatrial enlargement. (B) Short-axis cardiac magnetic resonance imaging T1 map at the mid-level, with the arrow pointing to the low septal T1 value of 704 ms. (C) T2∗ mapping. Red shading in the left ventricle represents a short T2∗ relaxation time. The white arrow points to the measured septal T2∗ relaxation time of 12 ms, consistent with iron overload. (D) Liver magnetic resonance imaging R2∗ sequence showing hyperintensity in the liver (red arrow) compared with other abdominal viscera, suggesting hepatic iron overload.

During hospital admission, TTE showed severe biventricular failure. His LV was severely enlarged (end-diastolic diameter 65 mm; normal range 41-55 mm) with severe generalized hypokinesis and an LVEF of 23%. He had a severely enlarged RV chamber size (basal diameter 67 mm; normal range 25-41 mm), and torrential tricuspid regurgitation due to tricuspid annular dilation and RV enlargement. Laboratory evaluation revealed a ferritin of 1,598 μg/L (previously 37 μg/L 3 years prior) and an iron saturation of >90%. His model for end-stage liver disease score was 38, driven predominately by a total bilirubin of 14.9 mg/dL and an international normalized ratio of 5.4. HFE genetic testing showed C282Y heterozygosity, with normal genotypes for S65C and H63D. Although this variant is present in 10% of the Caucasian population, it is not typically associated with clinically significant iron overload.^5^ CMR showed a septal native T1 value of 704.5 ms and a septal T2∗ relaxation time of 12 ms, compatible with severe IOC (Figure 1). There was no convincing myocardial late gadolinium enhancement, although these sequences were limited by motion. Liver magnetic resonance imaging (MRI) and elastography showed a mean liver stiffness of 4.2 kPa (stage 3-4 fibrosis) with a mean liver iron concentration of 8.3 mg of iron per gram of liver corresponding to significantly elevated iron overload.

During the patient's hospital stay, he continued to decompensate and developed new renal failure despite inotropic agents, iron chelation with deferasirox, and diuresis. Given his multiorgan involvement, he was deemed ineligible for combined liver and heart transplantation and unfortunately died after transitioning to palliative care.

## Case 2

A 56-year-old man with a history of cryptogenic cirrhosis presented to the emergency department with new-onset dyspnea and a 15-lb weight gain. His diagnosis of cirrhosis was made 2 years before this evaluation, after he was found to have anemia with subsequent work-up showing gastric antral vascular ectasia and esophageal varices. He had no significant history of alcohol use or obesity, and extensive laboratory evaluation, including autoimmune studies, alpha-1 antitrypsin, ceruloplasmin, and viral hepatitis, was unremarkable. Genetic testing for HH demonstrated heterozygosity for C282Y, with no mutations in S65C and H63D variants. Routine TTE obtained 9 months before the current evaluation showed an LVEF of 71% and normal LV and RV sizes. Coronary angiogram obtained at that time showed angiographically normal coronary arteries with no significant coronary artery disease.

During this admission, a repeat TTE demonstrated a newly reduced LVEF of 38% with a LV end-diastolic diameter of 59 mm and moderate global hypokinesis. Although his serum iron studies had historically demonstrated iron deficiency, his current laboratory work showed a ferritin of 1,658 μg/L (32 μg/L 1 year prior) with an iron saturation of >90%. Liver MRI showed iron overload with a T2∗ of 1.8 ms with an estimated iron content of 22.8 mg of iron per gram of liver. Subsequent CMR demonstrated late gadolinium enhancement in a nonischemic pattern, predominantly affecting the basal inferior, basal and mid-inferolateral, and basal anterolateral wall segments. It also showed a reduced native T1 value of 753 ms and a T2∗ relaxation time of 13 ms, consistent with IOC. Consequently, he was initiated on chelation therapy with deferoxamine and is currently undergoing evaluation for a combined heart-liver transplantation.

## Discussion

Cirrhosis leading to IOC in the absence of iron-loading conditions is a recognized entity in the literature, but few clinical cases have been reported with clinical congestive heart failure due to IOC.^6^^,^^7^ The 2 cases herein describe rapid-onset IOC in patients before liver transplantation, which has not been previously reported. Given the profound implications for morbidity and mortality and the possible impact on transplantation status, early detection of IOC is critical.

Stable serum iron levels are maintained when hepcidin binds to and degrades ferroprotin, the major iron exporter protein present in intestinal epithelial cells, the reticuloendothelial system, and hepatocytes. Reduced hepcidin synthesis in advanced liver disease results in increased iron absorption and release, with subsequent iron accumulation in multiple organs, resulting in increased ferroportin-mediated iron absorption and release.^3^ In a small series by O'Glasser et al,^8^ 14 patients without HH and grade 4+ hepatic iron deposition were referred for endomyocardial biopsy.^8^ Strikingly, 64% of these patients had evidence of cardiac hemosiderosis, which could not be reliably predicted by ferritin or transferrin saturation levels alone.

IOC may represent a significant contributor to morbidity and mortality in patients with cirrhosis. It was previously demonstrated by Kowdley et al^9^ that patients with hepatic iron overload experience lower post–liver transplantation survival rates than do those without, with cardiac causes of death accounting for 20% of mortality. Although our report highlights rapid-onset IOC, we hypothesize that subclinical or insidious IOC may affect a much larger proportion of patients with cirrhosis contributing to the high rates of cardiovascular dysfunction seen in this population.

Interestingly, both patients in our case series were heterozygous for HFE. In a case series by Eng et al^6^ of 3 patients with autopsy evidence of IOC but without clinical congestive heart failure before death, 2 were also found to be heterozygous for mutations in H63D. Similarly, Habib et al^7^ described a case of a woman with a heterozygous C282Y mutation who developed acute congestive heart failure 4 days after liver transplantation and was subsequently found to have IOC, although stress cardiomyopathy was also suspected to have contributed to her clinical decompensation. Together, this anecdotal evidence suggests that HFE heterozygosity may be a “second hit” in the setting of impaired hepcidin regulation leading to overt IOC, but future studies would be needed to further evaluate this.

There are presently no definitive guidelines for screening for IOC in patients with cirrhosis. A TTE can show evidence of dilation or systolic function, diastolic dysfunction, or restrictive filling patterns, and strain imaging may help detect subtle ventricular dysfunction. These findings are relatively nonspecific and are potential consequences of iron deposition on myocardial function. If there is any clinical suspicion for IOC, CMR should be obtained. The American Heart Association recommends further evaluation with CMR if there is clinical suspicion for IOC combined with a serum ferritin of >250 μg/L in men (or >200 μg/L in women) and a transferrin saturation of >45%.^10^ However, abnormal iron indices are highly prevalent in advanced liver disease, and the true risk of IOC is likely underappreciated. Treatment typically centers around chelation therapy and phlebotomy, though the evidence supporting these modalities is largely derived from primary iron overload (HH) and other secondary iron-loading conditions rather than cirrhosis. Iron removal is more effective when initiated in early disease, whereas reversal of advanced heart failure is often slow and incomplete. Moreover, patients with decompensated heart failure may not tolerate phlebotomy very well. These limitations highlight the importance of early recognition and sensitive screening to enable intervention before irreversible myocardial injury occurs.

Currently, the overall incidence of subclinical IOC in cirrhosis, the natural disease progression, and the utility of serial laboratory work and imaging for screening is not well characterized and requires further study. There is ample opportunity for a prospective CMR study of patients with cirrhosis, especially in those undergoing transplantation evaluations, where findings may have implications for organ eligibility. In addition, although strain findings are nonspecific, there may be utility in serial strain studies in IOC, and strain has never been studied in IOC secondary to cirrhosis.

It is important to acknowledge that decline in LV function may be multifactorial, and alternative etiologies of cardiomyopathy should always be considered, including ischemic, inflammatory, stress-induced, and high-output (cirrhotic) heart failure, and other infiltrative disorders. In the aforementioned cases, both patients' T2∗ values were only in the mild-to-moderately low range (10-20 ms). Although these findings are diagnostic of IOC, particularly in the context of the clinical presentation and laboratory results, an important limitation of these cases is the absence of endomyocardial biopsy to evaluate for alternative etiologies and to increase confidence in the final diagnosis.

## Conclusions

We describe 2 cases of rapid-onset IOC in the setting of non-HH cirrhosis, highlighting a highly morbid disease entity with a profound knowledge gap regarding the screening and treatment of this complication in patients with advanced liver disease. There is a need for prospective studies using cardiac imaging to elucidate the natural history of this disease in cirrhosis.

## Funding Support and Author Disclosures

The authors have reported that they have no relationships relevant to the contents of this paper to disclose.
